# Prevalence, Genomic Characterization, and Transmission Patterns of *Cronobacter* spp. in Low-Water-Activity Foods from Hunan Province, China

**DOI:** 10.3390/microorganisms14061320

**Published:** 2026-06-12

**Authors:** Fang Liu, Zhifei Zhan, Yating Ma, Wansi Zhang, Tianbing Lai, Shuai Chen

**Affiliations:** Hunan Provincial Center for Disease Control and Prevention (Hunan Academy of Preventive Medicine), Changsha 410005, China; hncdc922@163.com (F.L.);

**Keywords:** *Cronobacter* spp., *Cronobacter sakazakii*, low-water-activity foods, whole genome sequencing (WGS), core genome multilocus sequence typing (cgMLST), antimicrobial resistance, virulence factors

## Abstract

*Cronobacter* spp. are opportunistic foodborne pathogens that can cause neonatal meningitis, necrotizing enterocolitis, and sepsis. This study conducted a systematic contamination survey and whole-genome epidemiological analysis of 562 low-water-activity food samples in Hunan Province of China. The results showed an overall *Cronobacter* spp. detection rate of 41.99% (236/562), with spices exhibiting the highest contamination rate (60.06%), and with high-level contamination samples (>110 MPN/g) concentrated in this category. The 236 isolates comprised 6 species, 120 sequence types, and 39 clonal complexes, with *C. sakazakii* being the most frequently isolated species (64.83%) and high-risk clones ST4, ST1, ST148, and ST64 prevailing. Multiple virulence genes (*TraJ*, *fur*, *rcsAB*, *rpoS*) and antimicrobial resistance genes (*qnrS1*, *bla*TEM-1, *bla*CTX-M-55, *bla*LAP-2, *aac(3)-IId*, *aadA2*, *tet(A)*, *floR*, *mcr-9.1*, *sul2*) were detected. Core genome multilocus sequence typing (cgMLST) identified two clustering patterns: Cluster C, whose genetic clustering was consistent with transmission associated with potential common upstream raw materials across different brands and provinces, and Cluster G, whose clustering suggested potential persistent colonization in the production environment across multiple batches of the same brand. This study elucidates the contamination characteristics of *Cronobacter* spp. in low-water-activity foods from Hunan Province and provides a basis for WGS-based active surveillance and supply chain traceability.

## 1. Introduction

*Cronobacter* spp. are facultatively anaerobic, Gram-negative opportunistic pathogens belonging to the family Enterobacteriaceae [1]. The genus is currently divided into seven species and three subspecies, including *C. sakazakii*, *C. malonaticus*, *C. turicensis*, *C. muytjensii*, *C. condimenti*, *C. universalis*, and *C. dublinensis* [2]. Infections in immunocompromised individuals (such as the elderly and infants) can cause meningitis, necrotizing enterocolitis, septicemia, and neurological sequelae, with mortality rates ranging from 40% to 80% [3,4]. The pathogenicity and virulence factors vary among different species of this genus; all six species except *C. condimenti* are considered pathogenic [5,6]. Among them, *C. sakazakii* is the most frequently isolated and most virulent species [7]. Of note, infections in adults can also manifest as septicemia, osteomyelitis, and pneumonia [8,9].

*Cronobacter* spp. exhibit remarkable environmental tolerance and can survive for extended periods under acidic, hyperosmotic, and extremely low-water-activity conditions [10]. To date, contamination by *Cronobacter* spp. has been detected in a variety of food matrices, including powdered infant formula, spices, cereals, meat, raw vegetables, and rice and flour products [11,12,13,14,15,16]. However, systematic studies on its transmission routes, molecular epidemiological characteristics, and pathogenic mechanisms remain limited. Although whole-genome sequencing (WGS) has revealed the antimicrobial resistance and virulence factor profiles of foodborne *Cronobacter* spp. isolates from certain regions of China [16], the prevalence, resistance spectrum, and virulence characteristics of *Cronobacter* spp. in low-water-activity foods in Hunan Province have yet to be systematically elucidated.

This study focused on three categories of low-water-activity foods (*aw* < 0.85): spices, medicinal and edible foods, and cereal-based complementary foods for infants and young children. This classification is consistent with the water activity values reported for spices [17], medicinal and edible foods [18], and cereal-based complementary foods for infants and young children [19] in the literature. Although the low-water-activity of these foods theoretically inhibits microbial proliferation, the intrinsic desiccation tolerance of *Cronobacter* spp. enables their long-term survival in such matrices, thereby posing a potential risk. The consumer base for these foods is broad—spices are daily seasonings, while medicinal and edible foods and cereal-based complementary foods are particularly prevalent among infants, young children, and middle-aged and elderly populations in China, which include immunocompromised susceptible individuals.

Therefore, this study aims to systematically assess the contamination risk of *Cronobacter* spp. in low-water-activity foods from Hunan Province through strain isolation and identification, WGS, and antimicrobial susceptibility testing. The specific objectives are to: (1) determine the contamination status, species distribution, and dominant clonal lineages of *Cronobacter* spp. across different food categories; (2) analyze antimicrobial resistance phenotypes and their genetic determinants; (3) elucidate the distribution and functional characteristics of virulence genes; and (4) investigate the transmission patterns and potential contamination sources of the strains by combining core genome multilocus sequence typing (cgMLST) with epidemiological data. This study will provide a scientific basis for continuous monitoring, contamination traceability, and the development of effective public health prevention and control strategies in the low-water-activity food supply chain.

## 2. Materials and Methods

### 2.1. Strain Source, Collection and Preparation

As part of the national food safety risk monitoring program, a total of 562 low-water-activity food samples were collected from farmers’ markets, supermarkets, retail stores, and online shops across 11 prefecture-level cities in Hunan Province during 2023–2024. These samples included 338 spice samples (Pepper, Chili, Cumin, Sichuan pepper, Cassia, Star anise, Fennel, Bay leaf, Five-spice powder, Tsaoko, other), 110 samples of medicinal and edible foods (Jujube, Fox nut, Black sesame, Licorice, Kudzu root, Ejiao), and 114 samples of cereal-based complementary foods for infants and young children (Raw cereal-based complementary foods and Standard cereal-based complementary foods).

The samples were transported under sealed, cool, dry, and ambient temperature conditions. Upon arrival, they were stored by category in the sample room and tested within three days. Cross-contamination, environmental contamination, and any changes in the counts or growth capacity of intrinsic food microorganisms were strictly avoided throughout the process. Sampling coverage varied by year; some prefecture-level cities participated in both years, while others participated only in one year. Detailed information on all 236 isolates (species, ST, CC type, source, sampling location, and date) is provided in Supplementary Table S1.

### 2.2. Isolation and Identification of Cronobacter *spp.*

The isolation of *Cronobacter* spp. was performed by municipal Centers for Disease Control and Prevention (CDC) following the national standard GB 4789.40-2016 (updated to GB 4789.40-2024 in 2024) for qualitative testing and most probable number (MPN) enumeration [20,21]. The brief procedure was as follows: three portions each of 100 g, 10 g, and 1 g of samples were weighed and added to 900 mL, 90 mL, and 9 mL of sterile buffered peptone water, respectively, to prepare 1:10 sample homogenates for incubation. Subsequently, 1 mL of the enrichment culture was transferred to 10 mL of modified lauryl sulfate tryptose broth with vancomycin (mLST-Vm) and incubated. Following enrichment, the cultures were streaked onto two Enterobacter sakazakii chromogenic agar plates. Suspected colonies (green or blue-green) were picked and streaked onto tryptic soy agar (TSA) plates. Presumptive colonies were identified by biochemical tests or mass spectrometry.

Confirmation and in-depth analysis of the isolates were conducted at the provincial CDC. After all the isolates were sent to the provincial CDC, each isolate was re-confirmed using the VITEK 2 automated microbial identification system (bioMérieux, Marcy-l’Étoile, France). Subsequently, antimicrobial susceptibility testing as well as whole-genome sequencing and analysis were performed on all the confirmed isolates.

### 2.3. Antimicrobial Susceptibility Testing

Antimicrobial susceptibility testing was performed using the broth microdilution method with the AutoMic-i600 automatic antimicrobial susceptibility testing system (Autobio, Zhengzhou, China) according to the manufacturer’s instructions. A total of 23 antimicrobial agents from 10 classes were selected for testing based on recommendations from national and international surveillance networks (e.g., NARMS, CARSS, CHINET, and the Pathogen Identification Network), as well as guidelines from the Clinical and Laboratory Standards Institute (CLSI), the U.S. Food and Drug Administration (FDA), and the European Committee on Antimicrobial Susceptibility Testing (EUCAST).

The tested antimicrobial agents included: quinolones (ciprofloxacin, nalidixic acid); penicillins (ampicillin); cephalosporins (ceftazidime, cefotaxime, cefoxitin, cefepime, cefuroxime, cefazolin, ceftiofur); β-lactam/β-lactamase inhibitor combinations (ampicillin/sulbactam); carbapenems (ertapenem, imipenem, meropenem); aminoglycosides (gentamicin, amikacin); tetracyclines (tetracycline, tigecycline); amphenicols (chloramphenicol, florfenicol); polymyxins (polymyxin E, polymyxin B); and the dual folate antagonist combination antibiotic (trimethoprim/sulfamethoxazole).

The minimal inhibitory concentration (MIC) for each antimicrobial was determined and interpreted using a unified set of breakpoints compiled from the following publicly available standards: CLSI M100-S34, CLSI M45-A3, CLSI VET01-A4, FDA and EUCAST [22,23,24,25,26]. *Escherichia coli* ATCC 25922 and *Pseudomonas aeruginosa* ATCC 27853 were used as quality control strains. Isolates resistant to three or more classes of antimicrobial agents were defined as multidrug-resistant (MDR) strains [27]. To ensure transparency and reproducibility, the complete breakpoint table is provided in Supplementary Table S3.

### 2.4. Whole Genome Sequencing and Data Processing

Genomic DNA was extracted using the TIANamp Bacterial DNA Kit (Tiangen Biotech, Beijing, China) according to the manufacturer’s instructions. WGS was performed by Novogene Bioinformatics Technology Co., Ltd. (Beijing, China) using the Illumina NovaSeq 6000 platform (Illumina, San Diego, CA, USA) with 150 bp paired-end (PE150) sequencing technology. Raw reads were processed using Fastp to remove adapter sequences and low-quality reads, yielding clean data [28]. Clean reads were assembled using SPAdes v3.15.0 software with default parameters [29]. Sequencing quality was assessed by evaluating sequencing depth, genome coverage, Q20/Q30 values, and assembly statistics (number of contigs and N50 values). Only assemblies that met quality thresholds for contig count and single-base error rate were used for subsequent analyses. Detailed quality metrics for each isolate were provided in Supplementary Table S2.

### 2.5. Bioinformatic Analysis

The cleaned sequencing data were uploaded to the Microobench platform (Zhongwei Shuchuang Technology Co., Ltd., Beijing, China)—a pathogenic microorganism analysis workstation—for subsequent bioinformatics analysis. Multilocus sequence typing (MLST) was performed based on seven housekeeping genes (atpD, fusA, glnS, gltB, gyrB, infB, pps) using the PubMed ST database. Virulence genes were identified by alignment against the Virulence Factor Database (VFDB) [30], requiring sequence identity ≥ 90% and coverage ≥ 90%. Antimicrobial resistance genes were identified by alignment against the Comprehensive Antibiotic Resistance Database (CARD) [31] using the same thresholds. All of the above thresholds were the default parameters of the Microobench platform.

Pan-genome analysis was performed based on genome annotation files (GFF) using Panaroo (version 1.6.0). The core genome was constructed under strict mode (--clean_mode strict), and the core gene set was extracted using both the default core gene threshold and a threshold of 90% (--core_threshold 0.9). After obtaining the core gene alignment sequence (core_gene_alignment_filtered.aln), a maximum likelihood phylogenetic tree was constructed using IQ-TREE software under the GTR + G substitution model, with branch support evaluated by 1000 bootstrap replicates, to assess the overall evolutionary relationships of the 236 *Cronobacter* spp. isolates.

To investigate the genetic relatedness among isolates, cgMLST was performed. Based on cgMLST allele profiles, a phylogenetic tree was constructed using the single-linkage clustering algorithm implemented in the Microobench platform. A threshold of ≤10 allele differences was used to define genomic clusters [32,33]. By integrating sampling information (date, city, county, market, and sample type), the identified genomic clusters were further categorized into two descriptive categories: (C) common-source-supported clusters (isolates with high genomic similarity but lacking strong spatiotemporal links within the same cluster, suggesting potential common upstream sources) and (G) genomic clusters only (clusters based solely on genomic similarity without clear spatiotemporal or source associations, representing background lineages). It should be noted that this classification was descriptive and does not directly equate to confirmed transmission events, nor can it completely exclude the possibility of cross-contamination during sampling or laboratory processing.

Due to the limited number of isolates of the other five species (*C. malonaticus*, *C. turicensis*, *C. muytjensii*, *C. universalis*, and *C. dublinensis*), conducting cgMLST analysis and phylogenetic tree construction for them would not be statistically meaningful. Therefore, cgMLST was performed only for *C. sakazakii* in this study to elucidate the transmission patterns of its isolates.

## 3. Results

### 3.1. Isolation and Prevalence of Cronobacter *spp.*

The detection rate and contamination level of *Cronobacter* spp. were analyzed in 562 low-water-activity food samples from Hunan Province of China, with an overall detection rate of 41.99% (236/562). The samples included 338 spices, 110 medicinal and edible foods, and 114 cereal-based complementary foods for infants and young children, with positive isolation rates of 60.06% (203/338), 13.64% (15/110), and 15.79% (18/114), respectively. Among the 236 positive samples, the contamination levels of *Cronobacter* spp. showed a skewed distribution: 68.22% (161/236) had contamination levels below 10 MPN/g, 15.68% (37/236) had levels between 10 and 110 MPN/g, and 16.10% (38/236) had levels exceeding 110 MPN/g. Among the 38 food samples with contamination levels exceeding 110 MPN/g, spices accounted for the highest proportion (36 samples in total), including pepper (12), cumin (17), chili (3), Sichuan pepper (2), and fennel (2). In addition, high contamination levels were also detected in jujube (1 sample) among medicinal and edible foods and in one sample of raw cereal-based complementary food for infants and young children (Table 1).

### 3.2. Species Distribution and Dominant Clonal Lineages of Cronobacter *spp.*

Among the 236 *Cronobacter* spp. isolates, six species were identified based on PubMLST analysis. *C. sakazakii* was the most frequently isolated species, accounting for 64.83% (153/236), followed by *C. malonaticus* (14.83%, 35/236), *C. turicensis* (8.47%, 20/236), *C. muytjensii* (5.93%, 14/236), *C. dublinensis* (4.23%, 10/236), and *C. universalis* (1.69%, 4/236). A total of 120 sequence types (STs) were identified, belonging to 39 clonal complexes (CCs). *C. sakazakii* exhibited the highest genetic diversity, comprising 61 STs and 25 CCs, with the dominant STs being ST4 (13 isolates), ST1 (12 isolates), ST148 (11 isolates), and ST64 (11 isolates), and the corresponding dominant CCs being CC4 (13 isolates), CC1 (12 isolates), CC16 (11 isolates), and CC64 (11 isolates). For *C. malonaticus*, 24 STs were identified, with ST211 (5 isolates) being the most common, and the main clonal complex was CC200 (5 isolates). The remaining species were isolated in smaller numbers, with a more dispersed distribution of STs.

From the perspective of food sources, spices were the category with the largest proportion of *Cronobacter* spp. isolates in this study, with 203 isolates detected, followed by medicinal and edible foods (15 isolates) and infant cereals (18 isolates). Notably, *C. sakazakii* was most frequently detected in spices (126 isolates) and was also present in both medicinal and edible foods and infant cereals. In contrast, *C. muytjensii* and *C. universalis* were detected only in spices. The identification of these dominant sequence type clonal strains provided a critical framework for subsequent studies on the dissemination of associated resistance and virulence determinants (Table 2).

### 3.3. Virulence Gene Profiles and Key Determinants

The virulence gene repertoire of 236 *Cronobacter* spp. isolates was systematically analyzed according to the Virulence Factor Database (VFDB) classification (Table 3).

Regarding adherence, the Sfp fimbriae gene was detected in 4.24% of isolates, while the REPEC fimbriae gene, essential for host colonization, was found in only 1.27% of isolates.

For effector delivery systems, the Type VI Secretion System-II (T6SS-II) was present in all the isolates, suggesting its core role in interbacterial competition. The T6SS was detected in 50.42% of isolates, whereas the T6SS-III was identified in only two isolates (0.84%). The Type III Secretion System (T3SS) was also rare, with a detection rate of 0.84%.

In terms of immune modulation, capsule biosynthesis genes were present in all the isolates, facilitating bacterial persistence and immune evasion. LPS-associated genes were detected in only 1.27% of isolates.

For invasion, the *TraJ* gene, involved in virulence plasmid spread, was detected in 2.12% of isolates. All the isolates carried genes for peritrichous flagella, enabling motility and chemotaxis.

Among the regulatory genes, *fur* (involved in nutrient sensing and virulence expression) and *rcsAB* were present in all the isolates. The detection rate of *rpoS* was 99.58%, which was closely associated with desiccation tolerance.

### 3.4. Antimicrobial Resistance Phenotypes and Their Genetic Determinants

Antimicrobial susceptibility testing was performed on 236 *Cronobacter* spp. isolates, with the results summarized by antibiotic category in Table 4. Overall, *Cronobacter* spp. isolates remained largely susceptible to most tested antibiotics, with only a few isolates exhibiting resistance or intermediate phenotypes. Five isolates (2.12%) were MDR.

Quinolones: All the isolates were susceptible (99.58%) or intermediate (0.42%) to ciprofloxacin, with no resistant strains detected. The resistance rate to nalidixic acid was 0.85% (2/236). The *qnrS1* gene was detected in 1.27% of the isolates.

Penicillins and cephalosporins: All the isolates were fully susceptible (100%) to ceftazidime, cefotaxime, and cefepime. For cefoxitin, the intermediate rate was 30.93% (73/236) and the resistance rate was 5.08% (12/236). For cefazolin, the intermediate rate was 24.15% (57/236) and the resistance rate was 68.22% (161/236). For ampicillin, the resistance rate was 0.42% (1/236). For ceftiofur, the resistance rate was 0.42% (1/236). Detected resistance genes included *bla*CTX-M-55 (0.42%), *bla*TEM-1 (0.42%), and *bla*LAP-2 (0.84%).

β-lactam/β-lactamase inhibitor combinations and carbapenems: All the isolates were fully susceptible to ampicillin/sulbactam, ertapenem, imipenem, and meropenem.

Aminoglycosides: Gentamicin resistance was observed in 0.42% (1/236) of isolates, with an additional 0.42% (1/236) showing intermediate susceptibility. All the isolates were susceptible to amikacin. Detected resistance genes included *aac(3)-IId* and *aadA2*.

Tetracyclines: Tetracycline resistance was observed in 0.85% (2/236) of isolates. All the isolates were susceptible to tigecycline. Detected genes included *tet(A)* (0.42%).

Amphenicols: For chloramphenicol, the intermediate rate was 1.69% (4/236) and the resistance rate was 0.85% (2/236). For florfenicol, 29.24% (69/236) of isolates were susceptible, 62.71% (148/236) were intermediate, and 8.05% (19/236) were resistant. The *floR* gene was detected in 0.84% of isolates.

Polymyxins: For polymyxin E, the resistance rate was 2.54% (6/236). The mobile colistin resistance gene *mcr-9.1* was detected in 3.81% of isolates.

Dual folate antagonist combination antibiotic: Trimethoprim/sulfamethoxazole resistance was observed in 0.85% (2/236) of isolates. Detected resistance genes included *sul2* (0.84%), *dfrA12* (0.42%), and *dfrA17* (0.84%).

### 3.5. Phylogenetic Relationships of Cronobacter *spp.* Isolates Based on Core Genome Analysis

**Figure 1:**
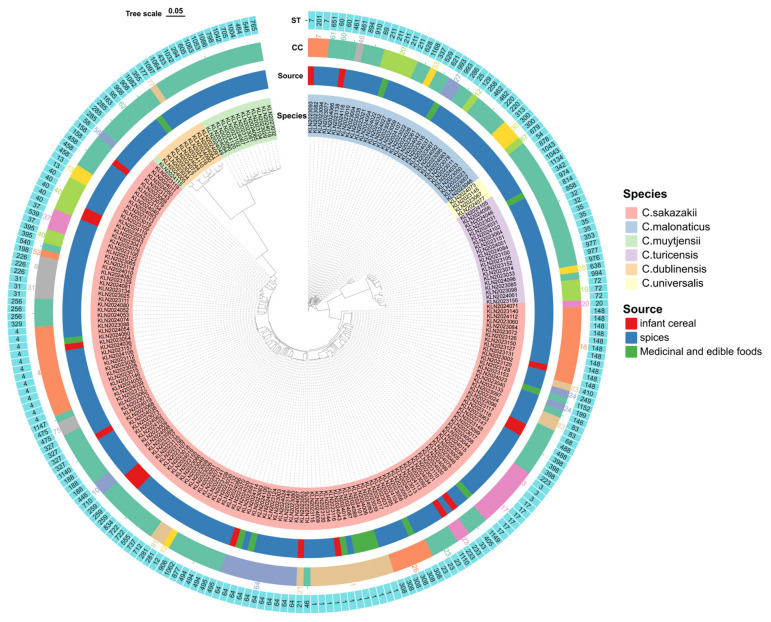
Maximum-likelihood phylogenetic tree of 236 *Cronobacter* spp. isolates based on core genome analysis. The tree was constructed using IQ-TREE with the GTR + G model and 1000 bootstrap replicates. Branch colors indicate species: *C. sakazakii* (red), *C. malonaticus* (blue), *C. muytjensii* (green), *C. turicensis* (purple), *C. dublinensis* (orange), and *C. universalis* (yellow). The outer rings represent sequence type (ST), clonal complex (CC), and food source (spices, medicinal and edible foods, infant cereals), respectively. Scale bar = 0.05 nucleotide substitutions per site.

To elucidate the overall evolutionary relationships among the 236 *Cronobacter* spp. isolates, a maximum-likelihood phylogenetic tree was constructed based on pan-genome analysis (Figure 1).

The phylogenetic tree clearly clustered the 236 isolates into six species-specific clades, consistent with the species identification based on MLST analysis. *C. sakazakii* formed the largest clade, comprising 153 isolates, followed by *C. malonaticus* (35 isolates), *C. turicensis* (20 isolates), *C. muytjensii* (14 isolates), *C. dublinensis* (10 isolates), and *C. universalis* (4 isolates).

### 3.6. Genomic Epidemiology and Transmission Patterns

**Figure 2:**
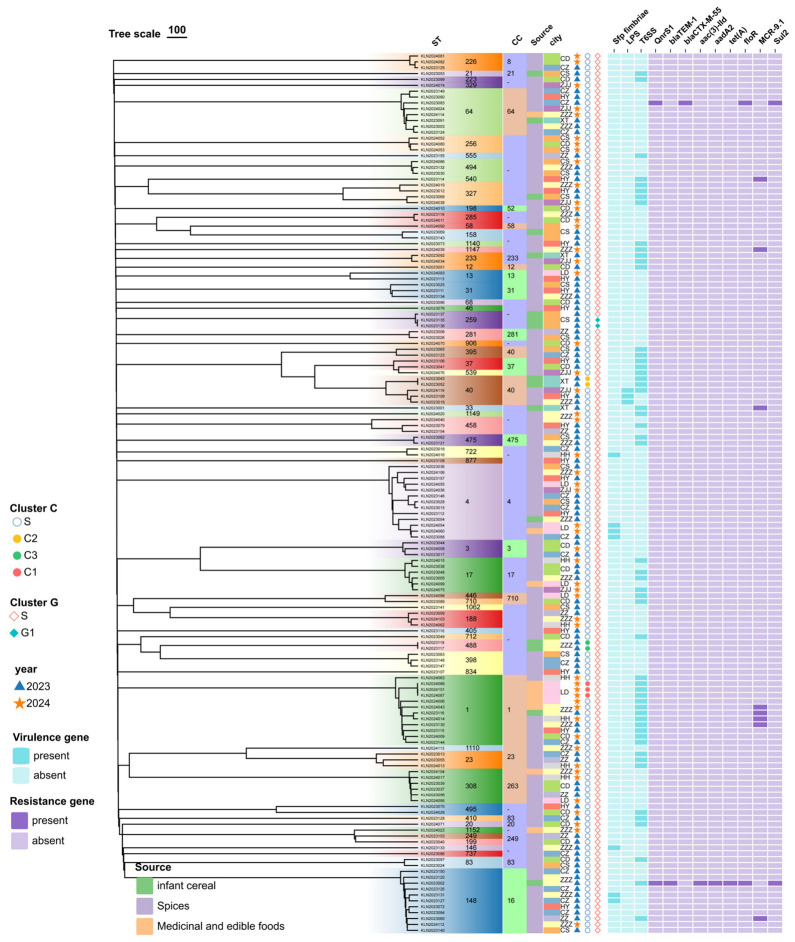
Phylogenetic relationships and distribution of key virulence and resistance genes among 153 °C. *sakazakii* isolates. The phylogenetic tree was constructed based on cgMLST analysis. Heatmaps show the presence (dark) or absence (light) of selected virulence genes (Sfp fimbriae, LPS, T6SS) and resistance genes (*QnrS1*, *bla*TEM-1, *bla*CTX-M-55, *aac(3)-IId*, *aadA2*, *tet(A)*, *floR*, *mcr-9.1*, *Sul2*). Clusters were labeled according to Table 5, with categories C and G distinguished by different shapes and colors.

CgMLST was performed to investigate the genetic relatedness among 153 *C. sakazakii* isolates recovered from different food products in Hunan Province, using a threshold of ≤10 allele differences to define genomic clusters. A total of 4 genomic clusters were identified and categorized into two types based on epidemiological evidence: common source-supported clusters (C) and genomic clusters only (G) (Table 5, Figure 2).

Common source-supported clusters (C). Three clusters (seven isolates) were assigned to this category, comprising isolates with high genomic similarity (0–2 allele differences) recovered from different time points or different sampling sites, but lacking strong spatiotemporal links (e.g., same-day and same-market exposure). These clusters suggested contamination from shared upstream sources in the supply chain.

Cluster C1 comprised three isolates collected from three different districts/counties in Loudi City within three days (3–5 September 2024; Ejiao brown sugar, Ejiao jujube, and ready-to-eat black sesame paste), with 1–2 allele differences.

Cluster C2 comprised two isolates collected from two different districts in Xiangtan City on the same day (24 May 2023; Lugang Cang and Beilaikeqin infant cereal products), with 0 allele differences.

Cluster C3 comprised two isolates collected from two different towns in Fenghuang County, Xiangxi Prefecture, on the same day (27 June 2023; Beileizhi and Tuxiaobei raw infant cereal products), with 0 allele differences.

Category G (genomic clusters only). Cluster G1 comprised two isolates with genomic similarity but no discernible epidemiological links and was assigned to this category, representing persistent or intermittent background lineages.

Cluster G1 comprised two isolates from Little Freddie brand infant cereal products in Changsha City (2 allele differences). Despite sharing the same manufacturer, they were assigned to Category G due to different sampling dates (12 days apart), production dates (38 days apart), and retail channels.

Among the four genomic clusters (C1–C3 and G1) identified by cgMLST, except for T6SS which was present in clusters C1 and C2, the distribution of other key virulence and antimicrobial resistance-associated genes (sfp fimbriae, LPS, *qnrS1*, *bla*TEM-1, *bla*CTX-M-55, *aac(3)-IId*, *aadA2*, *tet(A)*, *floR*, *mcr-9.1*, *sul2*) was sporadic across all the clusters, with no statistically significant lineage enrichment observed.

## 4. Discussion

### 4.1. Contamination Level of Cronobacter *spp.* in Low-Water-Activity Food Samples

The contamination level of *Cronobacter* spp. in 562 low-water-activity food samples from Hunan Province indicated that these foods serve as potential reservoirs of *Cronobacter* spp., which is consistent with the strong desiccation tolerance of this genus, particularly *C. sakazakii* [34]. Compared with similar studies in China and internationally, the detection rate in spices from Hunan Province (60.06%) was significantly higher than that in powdered spices from Nanning (25.0%) [12] and low-water-activity functional foods from Brazil (36.2%) [35], and also slightly higher than a previous report on retail spices in China (57.1%) [36]. However, due to differences in sample types and scales across these studies, accurate comparisons of detection rates were difficult, which also limits in-depth analysis of genetic diversity and transmission patterns. Regarding contamination levels, among the 38 high-risk samples exceeding 110 MPN/g, 36 originated from spices, indicating that this category not only has a high detection rate but also exhibits more severe contamination levels. In contrast, fewer high-level contamination samples were found in medicinal and edible foods and infant cereals. The samples in this study were collected from different pathways in Hunan Province, suggesting that the differences in contamination levels among different food categories may be associated with raw material sources, processing techniques, and storage conditions, the specific causes of which require further investigation in subsequent studies.

### 4.2. Species Distribution, Dominant Clones, and Pathogenesis of Cronobacter *spp.*

Based on PubMLST analysis, the 236 *Cronobacter* spp. isolates were identified and this result was consistent with the species identification based on the pan-genome maximum-likelihood phylogenetic tree.

A total of 120 STs belonging to 39 CCs were identified among all the isolates. *C. sakazakii* exhibited the highest genetic diversity, encompassing 61 STs and 25 CCs, with the dominant STs being ST4 (*n* = 13), ST1 (*n* = 12), ST148 (*n* = 11), and ST64 (*n* = 11). These were primarily sourced from spices and were also distributed in medicinal and edible foods as well as infant cereals. According to the PubMLST database, all four STs can be recovered from food, environmental, and clinical samples. Previous studies have shown that ST4, ST1, and ST64 are the predominant types of *C. sakazakii* in commercially available infant formula in China [37]; ST4, ST17, ST1, and ST64 are the dominant STs among clinical isolates [38]; while ST64, ST148, and ST4 are predominant in raw materials and production environments of infant formula factories [39]. Neonatal meningitis cases are primarily caused by *C. sakazakii* ST4 [40], and ST1 has been isolated from sputum samples of a newborn with severe pneumonia [41]. According to PubMLST records, an ST148 isolate was obtained from the blood sample of a 64-year-old patient in Denmark in 2009 [5]. Collectively, these findings indicate that different STs exhibit distinct clinical association profiles: ST4 is highly associated with neonatal meningitis and necrotizing enterocolitis; ST1 primarily causes respiratory and systemic infections in infants; ST148 tends to cause disease in immunocompromised elderly individuals; while ST64 is mainly detected in food products with relatively limited clinical reports of disease. For the other five species, the number of isolates was relatively small, with scattered ST distributions. These results suggest that *Cronobacter* spp. prevention and control strategies should move toward differentiation and precision.

### 4.3. Virulence Gene Profiles and Pathogenic Implications

In this study, a systematic virulence gene profiling analysis was performed on 236 *Cronobacter* spp. isolates, revealing the multi-layered pathogenic potential of this genus.

Adhesion and environmental persistence: The sfp fimbrial gene cluster exhibits species specificity, and strains carrying it show higher cytotoxicity [42]. Adhesion is not only the first step in host infection but also a core mechanism for the environmental persistence of *Cronobacter* spp.—*C. sakazakii* can form biofilms on the surface of infant formula production equipment, which is a key reason for its survival under extreme desiccation conditions and consequent recurrent contamination [43,44].

Effector delivery system: The type VI secretion system-II (T6SS-II) is present in all the isolates. The genome of *C. sakazakii* ATCC 12868 contains two functionally differentiated T6SS gene clusters: T6SS-1 is primarily involved in host pathogenicity, while T6SS-2 mainly mediates interbacterial competition [45,46]. T6SS exhibits species specificity, with highly cytotoxic isolates possessing a more complete T6SS gene cluster structure [42]. Moreover, T6SS gene clusters are located on mobile genetic elements, and structural variations among different species reflect ongoing genomic recombination and adaptive evolution [47].

Immune modulation and invasion: Capsule synthesis-related genes are universally present and serve as key factors for the long-term survival of this bacterium in dry environments. LPS can induce strong intestinal inflammation and disrupt intestinal barrier integrity [48]. *TraJ*, as a conjugative transfer regulatory protein, promotes the horizontal dissemination of virulence factor-carrying plasmids among populations, representing an important mechanism for the rapid spread of virulence factors [46,47].

Global regulation: The *fur* gene acts as an iron-responsive global regulator linking nutrient sensing with virulence expression. Studies have confirmed that *rcsAB* is a key regulator of *C. sakazakii* virulence [49], while *rpoS* is particularly critical for its desiccation tolerance in low-water-activity foods.

In summary, given that children, the elderly, and immunocompromised individuals are at high risk of infection by this bacterium in low-water-activity foods, further elucidation of its pathogenic mechanisms is of significant public health importance for the development of precise prevention and control strategies.

### 4.4. Antimicrobial Resistance Mechanisms and Clinical Implications

Antibiotic susceptibility analysis of 236 *Cronobacter* spp. isolates in this study indicated that this genus remains largely susceptible to most tested antibiotics, with a low rate of MDR. This finding is consistent with several recent studies—although *Cronobacter* spp. is widely distributed in foods and the environment, the overall level of acquired resistance remains limited [50,51]. Carbapenems (imipenem, meropenem, ertapenem), β-lactam/β-lactamase inhibitor combinations (ampicillin/sulbactam), and third- and fourth-generation cephalosporins (ceftazidime, cefotaxime, cefepime) all maintained complete susceptibility, indicating that these drugs can still serve as reliable treatment options for severe *Cronobacter* spp. infections. However, beneath the overall low resistance level, there are hidden concerns that warrant attention. Although the proportion of multidrug-resistant strains is low, their emergence indicates that the accumulation of resistance genes has begun to occur. Previous studies have reported that multidrug resistance has also been detected in *C. sakazakii* isolates from infant foods, and some clinical isolates can carry multiple resistance plasmids simultaneously [41,52]. Therefore, continuous monitoring of antimicrobial resistance trends in *Cronobacter* spp. is essential to prevent potential therapeutic challenges.

In this study, cefazolin showed a high resistance rate, and cefoxitin also displayed a moderate-to-high proportion of non-susceptibility, whereas the detection rates of acquired β-lactamase genes were extremely low. This phenotype-genotype “discordance” is not a true contradiction but rather reflects the intrinsic resistance characteristics of *Cronobacter* spp. mediated by chromosomally encoded AmpC-type β-lactamases. Pan-genome analysis has confirmed that the β-lactamase-encoding genes *bla*CSA and *bla*CMA are present in almost all *Cronobacter* spp. genomes [48,53]. These chromosomally encoded AmpC enzymes effectively hydrolyze first-generation cephalosporins and cephamycins but have limited hydrolytic activity against third- and fourth-generation cephalosporins, perfectly explaining why ceftazidime, cefotaxime, and cefepime maintain complete susceptibility.

In contrast, the phenotype and genotype for other classes of antibiotics showed good consistency. The tetracycline resistance rate was generally consistent with the detection rate of the *tet(A)* gene. The active efflux pump encoded by *tet(A)* is a classical mechanism of tetracycline resistance [54]. The chloramphenicol resistance rate corresponded well with the detection rate of the *floR* gene. The efflux pump encoded by *floR* can confer resistance to both chloramphenicol and florfenicol. The trimethoprim/sulfamethoxazole resistance rate could be explained by the combined carriage of *sul2* and *dfrA12/dfrA17* genes. Notably, the susceptibility profile of florfenicol exhibited a pattern distinctly different from other antibiotics—only a small proportion of isolates were susceptible, the majority were intermediate, and a notable fraction were resistant, yet the detection rate of the *floR* gene was extremely low. This severe phenotype-genotype mismatch is not a data anomaly but rather reveals the multi-layered nature of antimicrobial resistance mechanisms in *Cronobacter* spp.: chromosomally encoded multidrug efflux pump systems may become overexpressed under environmental stress, actively extruding florfenicol, a process controlled by global regulatory factors and independent of acquired resistance genes [55]; *Cronobacter* spp. may reduce membrane permeability by modifying outer membrane structures, thereby decreasing intracellular drug accumulation, manifesting as elevated MIC values or intermediate susceptibility in routine antimicrobial susceptibility testing [56]; and the interpretation of “intermediate” status based on current clinically derived breakpoint criteria should be approached with caution, as this status may represent an early signal of resistance evolution [57]. Transcriptomic studies have demonstrated that *Cronobacter* spp. can activate multidrug efflux systems in response to antibiotic stimulation by regulating chemotaxis-related genes [58]. Future studies should further elucidate the molecular basis of reduced susceptibility to florfenicol in this genus.

In this study, the *mcr-9.1* gene was detected in some isolates, yet the phenotypic colistin resistance rate was low, suggesting that the presence of this gene does not necessarily lead to high-level clinical resistance. Previous studies have indicated that the expression of *mcr-9.1* is regulated by multiple factors, including promoter mutations, insertion sequence-mediated regulation, and synergistic interactions with host lipid A modification genes, and that *mcr-9.1*-mediated resistance often manifests as low-level or heteroresistance, which is easily underestimated by routine MIC assays [55]. Therefore, relying solely on genomic sequencing may overestimate clinical risk, while exclusive dependence on phenotypic testing may overlook resistance gene reservoirs. This study did not assess the transcriptional level of *mcr-9.1* or the polymorphisms in its upstream regulatory region. Future studies should integrate transcriptomics and refined antimicrobial susceptibility testing to elucidate its regulatory mechanisms and dynamic changes under environmental stress, thereby improving the accuracy of antimicrobial resistance surveillance.

The antimicrobial resistance profile data from this study have direct implications for the empirical treatment of *Cronobacter* spp. infections. The high-risk populations for *Cronobacter* spp. infection are newborns and premature infants, which can cause meningitis, necrotizing enterocolitis, and bacteremia, with extremely high mortality rates. Based on the data from this study: (1) Carbapenems and third- and fourth-generation cephalosporins (completely susceptible) can be used as the first choice for empirical treatment of severe *Cronobacter* spp. infections; (2) Cefazolin should not be used for infections caused by this genus due to its high intrinsic resistance rate; (3) Colistin, as a last-line drug, should be used after comprehensive evaluation combining the antimicrobial susceptibility testing results and *mcr-9.1* gene screening. From the perspective of public health surveillance, existing epidemiological evidence suggests that low-water-activity foods are one of the important potential transmission vehicles for *Cronobacter* spp. Resistant strains in food production environments not only directly threaten susceptible populations but also may facilitate the further spread of mobile resistance genes within microbial communities along the food chain. The dissemination of *mcr-9.1* plasmids among microorganisms in food processing environments is of particular concern, as colistin is one of the last-resort options for treating multidrug-resistant Gram-negative bacterial infections.

### 4.5. Genomic Epidemiology and Transmission Patterns

This study identified four genomically clustered groups with high genetic relatedness (C1–C3 and G1) through cgMLST, which suggests the possible existence of two coexisting potential contamination transmission routes in the low-water-activity food supply chain of Hunan Province.

Category C clusters exhibited high genetic relatedness across different brands and provinces. By assessing the genetic relatedness among isolates to investigate possible transmission patterns, this pattern suggests the risk that different end-product manufacturers may share the same upstream raw material supplier or co-packing facility. Lu et al., in a survey of four powdered infant formula (PIF) factories in China, demonstrated that strains from the same production facility were phylogenetically more closely related than those from different facilities, with PIF residues, fluidized beds, and drying areas being the main positive contamination zones [59]. Gan et al., in a study of a PIF factory in Shaanxi Province, revealed close phylogenetic relationships among isolates from raw materials and production environment samples, suggesting cross-contamination between processing workshops and the external environment [39]. Together, these lines of evidence suggest that the traditional recall model based on “brand/manufacturer” may have limitations in addressing such potential cross-regional risks, and risk assessment needs to be validated through more in-depth ingredient-level traceability investigations.

Category G clusters: Hypothesis of persistent colonization in the production environment based on genetic relatedness. G1 clusters exhibited high temporal genetic relatedness within the same brand. This pattern suggests the possible existence of persistent colonization in the production environment, leading to intermittent contamination of different batches of products by the strains. Stevens et al., using cgMLST analysis of *C. sakazakii* isolated from a Swiss PIF factory over a 15-year period, directly demonstrated that the ST83 clonal group persisted in the same factory for more than 15 years, forming an evolving persistent population [60]. The molecular mechanisms supporting such persistence include biofilm formation, subpopulation development, and high tolerance to environmental stressors [61,62]. Given this, the public health implication of Category G clusters is that routine finished product batch testing may not be able to effectively identify the potential risk of persistent colonization in the production environment. Therefore, it is recommended to initiate more in-depth environmental monitoring programs targeting the relevant production facilities, including regular and systematic sampling and traceability investigations in key areas of the processing workshop, to verify whether specific environmental reservoirs exist.

The sporadic distribution of virulence and resistance genes in this study indicates that relying solely on cgMLST clustering or ST typing cannot reliably predict the resistance profile or virulence potential of a given strain. Future research urgently needs to integrate long-read sequencing and plasmid typing technologies to systematically characterize the transmission network of resistance plasmids within the *C. sakazakii* population, thereby providing targeted intervention evidence for source control.

## 5. Conclusions

In this study, a systematic WGS analysis of *Cronobacter* spp. in low-water-activity foods from Hunan Province revealed the distribution patterns, genetic diversity, and resistance and virulence characteristics of this pathogen in specific food matrices. The results showed an overall detection rate of *Cronobacter* spp. of 41.99%, with spices exhibiting the most severe contamination (60.06%) and serving as the main source of high-level contamination (>110 MPN/g). *C. sakazakii* was the most frequently isolated species (64.83%), and globally high-risk clinical clones such as ST4, ST1, ST148, and ST64 were widely prevalent. The study found that the isolates maintained 100% susceptibility to carbapenems and third-/fourth-generation cephalosporins, with a multidrug resistance rate of only 2.12%. Multiple resistance-associated genes (*qnrS1*, *bla*TEM-1, *bla*CTX-M-55, *bla*LAP-2, *aac(3)-IId*, *aadA2*, *tet(A)*, *floR*, *mcr-9.1*, *sul2*) were detected. The detection rate of *mcr-9.1* (3.81%) was higher than the phenotypic resistance rate (2.54%), suggesting a hidden transmission risk under gene silencing. Virulence genes (*TraJ*, *fur*, *rcsAB*, *rpoS*) covered adhesion, effector delivery, immune evasion, and global regulation, conferring strong pathogenic potential and environmental adaptability.

Two clustering patterns with differentiated interventions: Category C (cross-brand/cross-province strains with high genetic relatedness): This pattern suggests the possibility of a common source at the upstream raw material or supply chain level. It is recommended to conduct traceability investigations and validation across regional supply chains. Category G (strains from the same brand with high temporal genetic relatedness): This pattern suggests the possible existence of persistent colonization in the production environment. It is recommended to strengthen targeted environmental monitoring, zonal sampling, and cleaning and disinfection verification. The above assessments are intended to provide a reference for prioritizing subsequent risk assessment, sampling strategies, and traceability efforts. Their certainty needs to be comprehensively validated by integrating more epidemiological and environmental evidence.

This study provides a relatively comprehensive genomic epidemiological baseline for the precise risk assessment of *Cronobacter* spp. in low-water-activity foods from Hunan Province, China, to date, and establishes a scientific foundation for the implementation of WGS-based routine surveillance and supply chain traceability mechanisms.

## Figures and Tables

**Table 1:** Prevalence and contamination level of *Cronobacter* spp. in 562 low-water-activity food samples (Spices, Medicinal and Edible foods, and Infant cereals ^1^) from Hunan Province, China.

Food Type	Food Category	Analyzed Samples, *n*	Positive Samples, *n* (%)		No. of Positive Samples by Quantitative Methods by MPN/g Range		
					MPN < 10	10 ≤ MPN < 110	110 ≤ MPN
Spice	Pepper	69	62	(91.30)	37	13	12
	Chili	71	31	(43.66)	22	6	3
	Cumin	62	51	(82.26)	29	5	17
	Sichuan pepper	45	28	(62.22)	26	0	2
	Cassia	34	9	(26.47)	9	0	0
	Star anise	22	5	(22.73)	4	1	0
	Fennel	10	7	(70.00)	4	1	2
	Bay leaf	7	5	(71.43)	4	1	0
	Five-spice powder	5	2	(40.00)	2	0	0
	Tsaoko	5	2	(40.00)	2	0	0
	Other	8	1	(12.50)	1	0	0
	Total	338	203	(60.06)	140	27	36
Medicinal and Edible foods	Jujube	20	2	(10.00)	1	0	1
	Fox nut	1	1	(100.00)	1	0	0
	Black sesame	29	7	(24.14)	4	3	0
	Licorice	5	1	(20.00)	0	1	0
	Kudzu root	1	1	(100.00)	1	0	0
	Ejiao	54	3	(5.56)	3	0	0
	Total	110	15	(13.64)	10	4	1
Infant cereals	R-CBCF ^2^	54	7	(12.96)	2	4	1
	S-CBCF ^3^	60	11	(18.33)	9	2	0
	Total	114	18	(15.79)	11	6	1
	Overall	562	236	(41.99)	161	37	38

^11^C ^1^ Cereal-based complementary foods for infants and young children. ^2^ Raw cereal-based complementary food for infants and young children. ^3^ Standard cereal-based complementary food for infants and young children.

**Table 2:** Characteristics of dominant *Cronobacter* spp. clones isolated from low-water-activity foods (Spices, Medicinal and Edible foods, and Infant cereals).

Bacterial Species	Positive Samples, *n* (%)	Predominant ST (s)	Predominant CC (s)	Source
*C. sakazakii*	153 (64.83)	61 STs; ST4 (13), ST1 (12), ST148 (11), ST64 (11)	25 CCs; CC4 (13), CC1 (12), CC16 (11), CC64 (11)	Spices (126), Infant cereal (16), Medicinal and Edible foods (11)
*C. malonaticus*	35 (14.83)	24 STs; ST211 (5)	10 CCs; CC200 (5), CC7 (3), CC300 (3)	Spices (31), Infant cereal (2), Medicinal and Edible foods (2)
*C. turicensis*	20 (8.47)	13 STs; ST35 (4), ST72 (3)	2 CCs; CC1009 (3), CC359 (1)	Spices (19), Medicinal and Edible foods (1)
*C. muytjensii*	14 (5.93)	10 STs; ST1004 (1), ST1032 (1)	–	Spices (14)
*C. dublinensis*	10 (4.23)	9 STs; ST908 (2)	2 CCs; CC162 (1), CC177 (1)	Spices (9), Medicinal and edible foods (1)
*C. universalis*	4 (1.69)	3 STs; ST1043 (2)	–	Spices (4)
Total	236 (100)	120 STs	39 CCs	236

**Table 3:** Prevalence of virulence factors based on VFDB classification.

VFDB Category	Virulence Factor/System	Positive Samples, *n* (%)	Key Function
Adherence	Sfp fimbriae	10 (4.24)	Enhanced cytotoxicity
	REPEC fimbriae	3 (1.27)	Essential for host colonization
Effector delivery system	Type III Secretion System (T3SS)	2 (0.84)	Participate in host cell invasion and immunoregulation
	Type VI Secretion System (T6SS)	119 (50.42)	Species specificity
	Type VI Secretion System-II (T6SS-II)	236 (100)	Interbacterial competition
	Type VI Secretion System-III (T6SS-III)	2 (0.84)	Bactericidal function
Immune modulation	Capsule	236 (100)	Aid in bacterial persistence in the host and immune system evasion
	LPS	3 (1.27)	Help bacteria modulate host immune responses or evade immune surveillance
Invasion	*TraJ*	5 (2.12)	Spread of virulence plasmids
Motility	Peritrichous flagella	236 (100)	Motility and chemotaxis
Regulation	*fur*	236 (100)	Nutrient sensing and virulence expression
	*rcsAB*	236 (100)	Key regulatory factors of virulence
	*rpoS*	235 (99.58)	Desiccation tolerance

**Table 4:** Antimicrobial resistance phenotypes and genetic determinants by antibiotic usage category.

Antimicrobial Group	Antibiotic	Phenotype (*n* = 236)			Main Genetic Determinant(s)	Prevalence (%)
		N (%) of S	N (%) of I	N (%) of R		
Quinolones	Ciprofloxacin	235 (99.58)	1 (0.42)	0 (0.00)	*QnrS1*; *emrB; emrR*	1.27; 93.64; 20.33
	Nalidixic acid	234 (99.15)	0 (0.00)	2 (0.85)	*QnrS1*; *emrB; emrR*	1.27; 93.64; 20.33
Penicillins	Ampicillin	235 (99.58)	0 (0.00)	1 (0.42)	*bla*_TEM-1_; *bla*_LAP-2_	0.42; 0.84
Cephalosporins	Ceftazidime	236 (100.00)	0 (0.00)	0 (0.00)	–	–
	Cefotaxime	236 (100.00)	0 (0.00)	0 (0.00)	–	–
	Cefoxitin	151 (63.56)	73 (30.93)	12 (5.08)	*bla*_CTX-M-55_; *bla*_TEM-1_; *bla*CMA-1; *bla*CMA-2; *bla*CSA-1; *bla*CSA-2; *bla*_LAP-2_	0.42; 0.42; 5.08; 18.22; 48.73; 17.80; 0.84
	Cefepime	236 (100.00)	0 (0.00)	0 (0.00)	–	–
	Cefuroxime	232 (98.31)	4 (1.69)	0 (0.00)	*bla*_CTX-M-55_; *bla*_TEM-1_	0.42; 0.42
	Cefazolin	18 (7.63)	57 (24.15)	161 (68.22)	*bla*_CTX-M-55_; *bla*_TEM-1_; *bla*CMA-1; *bla*CMA-2; *bla*CSA-1; *bla*CSA-2; *bla*_LAP-2_	0.42; 0.42; 5.08; 18.22; 48.73; 17.80; 0.84
	Ceftiofur	235 (99.58)	0 (0.00)	1 (0.42)	–	–
β-lactam/β-lactamase inhibitor combinations	Ampicillin/sulbactam	235 (99.58)	1 (0.42)	0 (0.00)	*bla*_TEM-1_	0.42
Carbapenems	Ertapenem	236 (100.00)	0 (0.00)	0 (0.00)	–	–
	Imipenem	236 (100.00)	0 (0.00)	0 (0.00)	–	–
	Meropenem	236 (100.00)	0 (0.00)	0 (0.00)	–	–
Aminoglycosides	Gentamicin	234 (99.15)	1 (0.42)	1 (0.42)	*aac(3)-IId*; *aadA2*	0.42; 0.42
	Amikacin	236 (100.00)	0 (0.00)	0 (0.00)	–	–
Tetracyclines	Tetracycline	234 (99.15)	0 (0.00)	2 (0.85)	*tet(A)*	0.42
	Tigecycline	236 (100.00)	0 (0.00)	0 (0.00)	–	–
Amphenicols	Chloramphenicol	230 (89.15)	4 (1.69)	2 (0.85)	*floR*	0.84
	Florfenicol	69 (29.24)	148 (62.71)	19 (8.05)	*floR*	0.84
Polymyxins	Polymyxin E	0 (0.00)	230 (97.46)	6 (2.54)	*mcr-9.1*; *bacA*	3.81; 20.34
	Polymyxin B	0 (0.00)	236 (100.00)	0 (0.00)	*mcr-9.1*; *bacA*	3.81; 20.34
Dual folate antagonist combination antibiotic	Trimethoprim/ sulfamethoxazole	234 (99.15)	0 (0.00)	2 (0.85)	*sul2*; *dfrA12*; *dfrA17*	0.84; 0.42; 0.84

S, susceptibility; I, intermediate resistance; R, resistant.

**Table 5:** Characteristics of genomic clusters identified by cgMLST analysis.

Cluster	Category	No.	Alleles	Epidemiological Context	Interpretation
C1	Common source	3	1–2	LD city (three days, 3–5 September 2024); three different areas	Localized temporal cluster
C2	Common source	2	0	XT city (same day, 24 May 2023); two different areas	Localized temporal cluster
C3	Common source	2	0	ZZZ city (same day, 27 June 2023); two different areas	Localized temporal cluster
G1	Genomic	2	2	CS city (12 days apart, 5 July 2023 vs. 17 July 2023); offline vs. online	Potential persistent or intermittent contamination lineage

Abbreviations: LD, Loudi; XT, Xiangtan; ZZZ, Xiangxi Tujia and Miao Autonomous Prefecture; CS, Changsha.

## Data Availability

The data presented in this study are openly available in Genome Sequence Archive (GSA) at https://ngdc.cncb.ac.cn/gsa/ (accessed on 7 June 2026), reference number CRA042174.

## Supplementary Materials

The following supporting information can be downloaded at https://www.mdpi.com/article/10.3390/microorganisms14061320/s1. Table S1: Basic characteristics of 236 *Cronobacter* spp. isolates; Table S2: Sequencing and assembly quality metrics for 236 *Cronobacter* spp. isolates. Table S3: Breakpoints used for antimicrobial susceptibility testing of *Cronobacter* spp. isolates in this study.
